# A System of Practical Medicine

**Published:** 1887-01

**Authors:** 


A SYSTEM OF PRACTICAL MEDICINE. *By American Authors. Edited by* WILLIAM PEPPER, M.D., LL.D., *and* LOUIS STARR, M.D. *Volume V.* DISEASES OF THE NERVOUS SYSTEM. *Large octavo, pp. 1326. Philadelphia:* LEA BROTHERS & COMPANY, *1886. Chicago:* A. C. McCLURG & COMPANY.

This large and handsome volume forms a fitting conclusion to the greatest undertaking of its kind ever attempted in this country. The editor is to be congratulated on the success of an enterprise involving in its preparation such a length of time and the coöperation of so many competent writers. One unacquainted with literary work of this kind can form little conception of the extent of the task, and of the energy and talent required to push it to a successful completion.

This volume comprises forty-four articles by twenty-three authors, among whom we find Folsom, Hamilton, Jacobi, Lyman, Mills, Mitchell, Seguin, Spitka and others quite as eminent in the field of neurology. It would be invidious to single out any of the articles for special commendation or criticism. To adequately review each would greatly exceed the space at the command of this journal. The work is a collection of monographs by men specially qualified to deal with the subject assigned them. The value of the book as a whole depends on the value of each essay. This method of producing a book has some positive advantages, in that much talent can be brought to bear on each subject. So far as the essays themselves are concerned, they would be just as valuable published as separate communications in current periodicals. Then the effect of reading a work of this kind is to produce an impression of distinctness which the subject does not naturally possess. This is to a certain extent overcome by the admirable introductory articles by Dr. Seguin on the "General Semeiology of Diseases of the Nervous System; Data of Diognosis" and "The Localization of Lesions of the Nervous System."

The work of the editor in defining the extent of each subject, as well as in classifying the various disorders, has been well done, though it is here that one might find fault, if anywhere. For example, the "Disorders of Speech" are considered in seven pages, while "Catalepsy" has twenty-five assigned to it. The relative importance and frequency of the disorders scarcely merit this difference. The work, as a whole, is a contribution to the literature of nervous diseases of which the American neurologists may well be proud.

